# Our New York Letter

**Published:** 1890-05

**Authors:** 


### OUR NEW YORK LETTER.

Dr. Jarvis, Professor of Laryngology at the New York University Medical College, in speaking at a recent clinic, of the treatment of hypertrophied tonsils, pronounced against the employment of cold water and astringent gargles for the control of hemorrhage that sometimes occurs after tonsillotomy; the teaching of Sir Morrell MacKenzie to the contrary notwithstanding. He claims that the fluid has a tendency to wash away the blood clots as rapidly as they are formed. In such cases, he urges absolute rest to promote clotting and to guard against vomiting.

He also condemns the employment of cocaine for the production of local anæsthesia in the removal of tonsils, explaining his reason on the grounds that the anæsthetic effect of cocaine is necessarily incomplete, being superficial in character, and the sense of an imaginary foreign body in the pharynx, which cocaine gives, may excite uncontrollable vomiting and thereby endanger the life of the patient by offering a mechanical interference to the clotting of the blood. Tonsils which show, from their gross appearances, a disposition to bleed should be removed by his wire snare ecraseur. Hemorrhagic or hard tonsils can be recognized by the sense of firmness imparted to the finger, and by their opacity and irregular contour. Very large tonsils when removed by means of the tonsilotome, should not be cut too close to their attachment lest hemorrhage be thereby provoked by section of larger arteries which ramify about the tonsil's base. He shows a preference for Matthieu's tonsilitome, for the reason that the harpoon attached to this instrument in seizing and retaining the excised tonsil prevents the possibility of the growth becoming detached and choking the patient. The bistoury he claims to be a relic of medieval surgery, which should be relagated to the confines of the dissecting room. Most of the recorded fatal hemorrhages that occur after tonsilotome have been the result of the use of the bistoury in the hands of even the most skillful surgeons. In the use of anæsthetics, which he employs onl ywhen operating upon unmanageable children, he has a decided preference for nitrous oxide gas, since consciousnes promptly returns under its use, and the danger of blood entering the larynx which is present whon chloroform is used, is obviated. When tonsils show signs of marked congestion he first exsanguinates them by the employment of appropriate gargles and sprays before undertaking their removal, so as to ensure complete protection against tonsillar hemorrhage.

Prof. Delafield, of the College of Physicians and Surgeons, recommends the following as the most reliable combination he knows of for a delated and weak heart:

℞ Ext. digitalis, fluidi, ♏ij.

Ext. convallareæ, fluidi, ♏xx.

Kali iodidi, gr. v.

To be taken as a dose three times a day after meals.

Digitalis strengthens the action of the left ventricle and convalaria of the right. He has given this combination in so many different forms of heart trouble and has found it so effectual in the case of a weak and dilated heart that he has come to recommend its use for such a condition.

Dr. Munde's treatment for chronic endometritis is as follows:

The uterine cavity having been first mopped clean with cotton applications, a conical tampon of absorbent cotton, soaked in a solution of equal parts of persulphate of iron and glycerine, is slipped into the uterine cavity on a slide application and permitted to remain there, a string being attached to it so that it can be removed when necessary. Another cotton tampon dusted with iodoform is placed against the cervix, and the vagina is tamponed with several more pledgets similarly deodorized. Both the vaginal and intra-uterine tampons should be removed in forty-eight hours, or if saturated before that time as soon as this fact is noticed. The patient is then put to bed and kept in a recumbent position with an ice bag on the hypergastrium until the tampons are removed. Should the patient experience a great deal of pain, morphine suppositories should be used according to the amount of pain present. In about two to four days after the operation the patient may be allowed to leave her bed, using hot two per cent. carbolized douches two or three times a day, and she is directed to present for further treatment within a week after the operation.

The subsequent treatment consists in the introduction into the uterus, of compound tincture of iodine on cotton wrrpped applicators, or of a favorite combination of Dr. Munde's, iodoform and alum, five grains of each in gelatine coated pencils, pushed into the uterus with a syringe.

He states that where these precautions were carried out, he has never seen any inflammatory reaction or disagreeable symptom follow this line of treatment.

Prof. J. A. Wyeth, of the New York Polyclinic, recently performed two amputations at the hip joint, at the hospital of the above named school, both patients making a good recovery· Dr. Wyeth, at a recent clinic, resected the stump of the humerus in a boy seven years of age, under cocaine anaesthesia. A conical shaped piece of bone about an inch in its long diameter, projected from the stump. He stated that he regarded this growth as a proliferation of bone cells, proceeding from the stump, and that after amputation of a limb in a child, such a condition frequently followed, demanding resection of the stump, which was necessary in this case. An Esmarch's bandage was drawn tightly around the shoulder of the amputated arm, and thirty minims of a 4 per cent. solution of cocaine were ehen injected deep into the tissues that surrounded the portion of bone to be removed. The soft parts were drawn up by means of a retractor, and with two or three strokes of the saw the conical shaped projection of bone was removed. The patient did not expererience the slightest pain or discomfort during or immediately following the operation. The flaps were again adjusted in position and healing took place by first intention.

